# Metformin and sodium dichloroacetate effects on proliferation, apoptosis, and metabolic activity tested alone and in combination in a canine prostate and a bladder cancer cell line

**DOI:** 10.1371/journal.pone.0257403

**Published:** 2021-09-27

**Authors:** Katharina Klose, Eva-Maria Packeiser, Petra Müller, José Luis Granados-Soler, Jan Torben Schille, Sandra Goericke-Pesch, Manfred Kietzmann, Hugo Murua Escobar, Ingo Nolte

**Affiliations:** 1 Small Animal Clinic, University of Veterinary Medicine Hannover, Foundation, Hannover, Germany; 2 Division of Medicine Clinic III, Hematology, Oncology and Palliative Medicine, University of Rostock, Rostock, Germany; 3 Institute for Pharmacology, Toxicology and Pharmacy, University of Veterinary Medicine Hannover, Foundation, Hannover, Germany; 4 Reproductive Unit-Small Animal Clinic, University of Veterinary Medicine Hannover, Foundation, Hannover, Germany; 5 Comprehensive Cancer Center Mecklenburg-Vorpommern (CCC-MV), Campus Rostock, University of Rostock, Rostock, Germany; Bauer Research Foundation, UNITED STATES

## Abstract

An important approach in tumor therapy is combining substances with different action mechanisms aiming to enhance the antineoplastic effect, decrease the therapeutic dosage, and avoid resistance mechanisms. Moreover, evaluating compounds already approved for the treatment of non-neoplastic diseases is promising for new antineoplastic therapies. Sodium dichloroacetate (DCA) reactivates oxidative phosphorylation in the cancer cell mitochondria, reducing apoptosis resistance in cancer cells. Furthermore, metformin inhibits the proliferation of tumor cells and CD133+ cancer -stem-like cells. In the present study, we evaluated the independent and synergistic effect of metformin and DCA on the metabolic activity, cell proliferation, and apoptosis of a canine prostate adenocarcinoma (Adcarc1258) and a transitional cell carcinoma cell line (TCC1506) in comparison to a primary canine fibroblast culture. Determining metformin uptake in tumor cells was performed by quantitative HPLC. Depending on the dosage, metformin as a single agent inhibited the metabolic activity and cell proliferation of the tumor cells, showing only minor effects on the fibroblasts. Furthermore, 1 mM metformin increased apoptosis over 96 h in the tumor cell lines but not in fibroblasts. Additionally, metformin uptake into the tumor cells *in vitro* was measurable by quantitative HPLC. Synergistic effects for the combination therapy were observed in both neoplastic cell lines as well as in the fibroblasts. Based on these results, metformin might be a promising therapeutic agent for canine urogenital tumors. Further studies on kinetics, toxicology, bioavailability, and application of metformin in dogs are necessary.

## Introduction

Canine prostate adenocarcinoma and transitional cell carcinoma are characterized by a local and invasive growth pattern and are clinically difficult to differentiate in dogs. Both tumors are associated with a poor prognosis due to late diagnosis, metastatic and invasive behavior, and limited therapeutic options [1–3]. Moreover, canine prostate adenocarcinoma is refractory to androgen deprivation therapy, which is the first-line therapy for prostate cancer in humans [4, 5]. Therefore, identifying and testing new therapy options are crucial. Tumor therapy in veterinary medicine focuses on the quality of life therapeutic benefit for patients.

Growing evidence indicates that cancer metabolism is a promising field for the development of new therapeutic approaches [6]. Independent of the tissue oxygen supply, tumor cells gain their energy through lactate fermentation, this phenomenon being described as the Warburg effect [7, 8]. During cellular respiration, reactive oxygen species (ROS) accumulate in the mitochondrion and depolarize the mitochondrion membrane. Consequently, voltage-gated channels are opened and pro-apoptotic factors are released. The missing release of pro-apoptotic factors is one possible explanation behind the resistance of tumor cells to apoptosis [9, 10]. Furthermore, lactate produced by tumor cells can disrupt the T-cell-metabolism and antigen presentation by dendritic cells, enabling the tumor cells to escape immune surveillance [11–13]. Considering the importance of lactate fermentation for cancer cells, it has been used in the development of metabolic-related targeted therapies [13–16].

Molecular weight of the small molecule sodium dichloroacetate (DCA) is 151 g/mol [17]. In addition, DCA is readily bioavailable after oral intake [17, 18]. As DCA is especially effective in cells with mitochondrial defects, it is used for treating disorders like congenital lactic acidosis [19], diabetes [19, 20], and pulmonary hypertension [21]. In the mitochondrion, DCA inhibits the pyruvate dehydrogenase kinase (PDK) while indirectly activating the pyruvate dehydrogenase (PDH) through inhibition of PDK- induced PDH phosphorylation [22, 23]. PDK inhibition and PDH activation induced by DCA can reverse the Warburg effect in tumor cells [24]. DCA exposure decreased phosphorylated PDH- and lactate levels in canine prostate adenocarcinoma and transitional cell carcinomas *in vitro* [23]. Moreover, Harting et al. demonstrated that canine cancer cell lines exposed to 10 mM DCA display an increased ROS production and reduced survival [25]. However, considering the reported achievable DCA plasma concentrations (0.3–3 mM) in humans [26] and dogs [27] as well as the reported slow plasma clearance of dogs [28], doses employed in the study by Harting et al. would probably be associated with severe side effects *in vivo* [25]. The ability of DCA to reverse apoptosis resistance in cancer cells and to sensitize them to other substances makes DCA interesting for combination therapy approaches, as a combination could reduce the required DCA doses.

Metformin is a biguanide with a small simple chemical structure, which is easy to synthesize. It has been used in human medicine for more than 60 years as a first-line therapy for type 2 diabetes [29]. Epidemiological studies reported a reduced hazard ratio for cancer, and prolonged survival times in diabetic patients after chronic metformin application [30–32]. Metformin induces two different types of cell death: Caspase-dependent and poly (ADP-ribose) polymerase (PARP)-dependent. Moreover, its application is not associated with cytotoxic effects in non-malignant mammary epithelial cells [33]. *In vivo* studies in mice showed the potential of metformin to reduce tumor growth of mammary carcinoma xenografts [34–37]. Furthermore, since metformin induces G0/G1 phase cell-cycle arrest, it especially reduces tumor stem cells and cell proliferation [34–38]. This effect makes metformin a very attractive agent for combination therapy with apoptotic inducers like DCA [35].

This study aimed to investigate the effect of metformin on the metabolic activity and cell proliferation of canine carcinoma-derived tumor cell lines. In addition, we investigated whether combining DCA and metformin has synergistic effects on the examined tumor cells, allowing a dose reduction in DCA. Furthermore, we investigated the effect of DCA and metformin on the tumor cell lines in comparison to that observed in non-malignant cells.

## Material and methods

### Tumor cell lines and cell culture

Two canine cell lines were used for the experiments: TihoDProAdcarc1258, abbreviated as Adcarc1258, derived from prostate adenocarcinoma [39, 40] and TihoDUrtTCC1506, abbreviated as TCC1506, established from a transitional cell carcinoma from female bladder tissue [25, 40]. Both cell lines were established and characterized in the Small Animal Clinic, University of Veterinary Medicine Hannover, Foundation, Hannover, Germany [39, 40]. The cells were regularly cultured with 5 mL medium 199 (Gibco™, Thermo Fisher Scientific GmbH, Darmstadt, Germany) including 10% fetal calf serum (FBS Superior, Biochrom GmbH, Berlin, Germany), and 2% penicillin-streptomycin (Biochrom GmbH) in 25 cm^2^ flasks (TPP, Techno Plastic Products AG, Trasadingen, Switzerland). The cell lines were tested for mycoplasma contamination by PCR protocol as previously described [41–44] and were confirmed to be mycoplasma-free. The incubation conditions were 37°C and 5% CO₂ in humidified air. Twice a week, the cells were detached with TrypLE™ Express (Gibco™, Thermo Fisher Scientific GmbH) and passaged in accordance with their individual proliferation rate and growth behavior.

### Fibroblast and myocytes isolation

A five-year-old male German Great Dane was presented to the Small Animal Clinic of the University of Veterinary Medicine Hannover in 2020 and euthanized because of a stomach rotation. Sampling was carried out 7 h after euthanasia in agreement with the owners. A six-year-old female Labrador Retriever was presented at the Small Animal Clinic of the University of Veterinary Medicine Hannover in 2021 due to dystocia. A medically indicated caesarean section and subsequent uterus resection (Sectio porro) were performed. Uterine myocytes, abbreviated as K9UtMyo1, were isolated from uterine tissue samples in agreement with the owner. Consequently, ethical approval was not required (German Animal Welfare Act, §7). To obtain a primary fibroblast culture, small pieces of tissue from the subcutis of the oral mucous membrane were resected from the German Great Dane after aseptic preparation. The uterine tissue sample was stored in isotonic sodium chloride solution overnight. The next day, the myometrium was macroscopically dissected from the remaining tissue and the obtained small myometrial and subcutis samples were immediately transferred to HANK`s transport medium (Hank`s salts, Biochrom GmbH). In a Petri dish, the tissue pieces were then cut into 1–2 mm cubes with a sterile scalpel. Subsequently, the fragments were enzymatically digested under constant mixing at 37°C and 5% CO₂ for 24 h. The digestion medium contained 0.2% collagenase (Serva Electrophoresis GmbH, Heidelberg, Germany) and 2% penicillin-streptomycin (Biochrom GmbH) in DMEM/HAM`s F-12 (PAN-Biotech GmbH, Aidenbach, Germany). For the fibroblast isolation, the enzymatically digested tissue fragments were additionally dissociated in the GentleMacs™ dissociator (Miltenyi Biotec B.V. & Co. KG, Bergisch Gladbach, Germany) for one minute. After dissociation, the fibroblast and myocyte cell suspensions were centrifuged at 1,000 x-g and 20°C for 10 min and the supernatant was removed. Then the cell pellets were washed in fibroblast cell culture medium. The culture medium consisted of 1:1 DMEM/HAM`s F-12 (PAN-Biotech GmbH), 2% streptomycin/penicillin (Biochrom GmbH) and 10% fetal calf serum (Biochrom GmbH). The cells were transferred in a 25 cm^2^ flask (TPP, Faust Lab Science GmbH, Klettgau, Germany). The fibroblasts and K9UtMyo1 were incubated under the conditions described in cell culture and subcultured in accordance with their slower growth kinetics.

### Fibroblasts and myocytes detection

For this purpose, the fibroblasts and myocytes were seeded in a 96 well plate of 7,500 cells/well and incubated for five days. After incubation, the cells were fixed with 4% paraformaldehyde for 20 min and then permeabilized with 0.2% Triton X-100 (Merck KGaA, Darmstadt, Germany) for 20 min and blocked with 1% BSA for 30 min, both at room temperature. After blocking, fibroblasts were incubated overnight at 4°C with primary antibodies (S1 Table). Primary antibody binding of the myocytes was carried out for 2 hours at room temperature including isotype control. Species reactivity for the dog was either declared by the manufacturer`s protocol or based on the literature [45–49]. Afterwards, the cells were washed with PBS and incubated with the fluorescent-dye conjugated secondary antibodies for 1 h at room temperature. The cell nuclei were stained for 3 min with 4,6-diamidino-2-phenylindole 1/1000 (DAPI) (Merck KGaA). Fluorescent labeling was studied under an inverse microscope DMI6000 B in 100-x magnification (Leica Microsystems GmbH, Wetzlar, Germany).

### Compounds

Dichloroacetic acid (Merck KGaA) was used to prepare a 1 M stock solution of DCA every four weeks and stored at 4°C. Different concentrations and dilution series from 0.1–20 mM DCA were prepared immediately before each experiment. The purchased dichloroacetic acid solution is given with a specific concentration of 1.547 g/mL. For the preparation of a 1 M DCA stock solution, 0.827 mL of the dichloroacetic acid stock solution was diluted in 2 mL distilled water. Subsequently, the pH was adjusted to pH 7 by titration with sodium hydroxide. The solution was filled up with distilled water to a final volume of 10 mL. Afterwards, the obtained DCA stock solution was sterile filtrated and stored at 4°C.

Metformin solution was always freshly prepared on the day of application. To prepare a 100 mM stock solution, 82.8 mg metformin hydrochloride (BIOMOL GmbH, Hamburg, Germany) were dissolved in 5 mL cell culture medium and sterile filtrated as well.

### Cell count analysis

The number of cells was determined after exposure to DCA, metformin or combinations of both substances. For this purpose, the cells were seeded in triplicate with a cell density of 5*10⁵ cells per well in six well plates (TPP, Faust Lab Science GmbH) in 3 mL medium. After 12 h of incubation, the cells were exposed to metformin (0.1, 1, and 5 mM, respectively), DCA (3, 5, and 10 mM, respectivley) or both substances for 48 or 96 h. After exposure, the cells were washed with 500 μL PBS and detached with 500 μL TrypLE™ Express (Gibco™, Thermo Fisher Scientific GmbH) per well. Subsequently, the cells were counted via automated Cellometer™ Auto T4 (Nexcelom Bioscience LLC., Lawrence, MA, USA). The untreated control was set at 100% as reference value.

### Evaluation of DCA and metformin synergistic effects on metabolic activity

To identify synergistic effects of DCA and metformin in multiple-dose combinations (DCA: 0.1–20 mM; metformin: 0.0001–10 mM), the *in vitro* checkerboard method was used as previously described [50, 51]. Herein, tumor and fibroblasts cells were seeded in a 96 well plate (TPP, Faust Lab Science GmbH) with a cell density of 7,500 cells/well. After 12 h, the medium was exchanged. A dilution series was produced for DCA and metformin. Concentrations for DCA were in a range between 0.1–20 mM, and for metformin, between 0.0001–10 mM. The cells in each well were exposed to 200 μL of a unique combination treatment for 48 or 96 h. An untreated control served as a reference. Metabolic activity was measured after exposure to the MTS-Assay (CellTiter96® Aqueous One Solution Assay, Promega GmbH, Walldorf, Germany) in accordance with the manufacturer`s instructions. The included tetrazolium salt was reduced by vital cells to formazan, which was measured photometrically at 490 nm using a Synergy 2 plate reader (BioTek Instruments GmbH, Bad Friedrichshall, Germany). Subsequently, the metabolic activity of the cells was normalized to the untreated controls. Each experiment was performed six times independently.

The Bliss independence model was used to determine synergistic effects between two substances with different mechanisms of action as previously reported [52, 53]. The model compares the predicted effect (E_AB,P_) of the single substance application of drug A (E_A_) and drug B (E_B_) with the observed effect (E_AB,O_) of the combined application. The predicted effect was calculated with the following formula:

$$E_{AB,P}=E_{A}+E_{B}-E_{A}E_{B}$$

The Bliss value was calculated by subtracting the predicted effect (E_AB,P_) from the observed effect (E_AB,O_). This allowed the distinction between synergistic effects (Bliss value >0), additive effects (Bliss value = 0), and antagonistic effects (Bliss value < 0). To further evaluate the synergistic effect and its biological relevance, differences between the metabolic activity of the combinations and the metabolic activity after separate application of the two substances were analyzed with Dunnett`s test for multiple comparisons, and confidence levels were set at 95% (p <0.05). Only when the metabolic activity of the combined application had significantly decreased in comparison to the respective single applications of DCA and metformin along with a Bliss value > 0 was a biological relevance of the synergistic effect of both substances assumed.

### Quantitative measurement of metformin in cell culture medium and cell pellet by HPLC

Cell lines were seeded in triplicate in six well plates with 5*10⁵ cells/well. After an adherent period of 12 h, the cells were treated with 0.1 mM metformin for 48 h. After exposure, the supernatant was stored at -20°C. The cells were harvested and counted as described for cell count analysis. Afterwards, the cell suspension was centrifuged at 4°C by 153 xg for 10 min. The supernatant was removed and the cell pellets were stored at -20°C. The high-performance liquid chromatography (HPLC) was conducted with the autosampler (508, Beckmann Lab, Munich, Germany) on a separation column LiChroCART® 250–4, LiChrospher® 100 CN, 5 μM (Merck KGaA) with a UV detector (166, Beckmann Lab) at a wavelength of 254 nm. Prior to this step, the guard column (LiChroCART® 4–4, LiChrospher® 100 CN, 5 μm) had been installed in the HPLC-system. Both columns were placed in the column oven SpH 99 (Spark Holland B.V., Emmen, the Netherlands) at 40°C to perform the measurement. The mobile phase consisted of 15% acetonitrile (Carl Roth GmbH & Co. KG, Karlsruhe, Germany) and 85% phosphate buffer, which, in turn, consisted of 0.05 g/L potassium dihydrogen phosphate (Merck KGaA) and 1.13 g/L disodium hydrogen phosphate dihydrate (Merck KGaA). By adding NaOH (Merck KGaA), the mobile phase was adjusted to a pH of 8.0 and then degassed by the Emmi -20HC ultrasonic bath (EMAG AG, Mörfelden-Walldorf, Germany). The flow rate was 0.7 mL/min and the pressure was 12.4 mPa. For performing HPLC analysis of metformin concentration in cell culture medium and cell pellets, the method of the external standard was used. For this purpose, a stock solution of metformin (1 mg/mL metformin hydrochloride (BIOMOL GmbH)/destilled water 1:1) was prepared freshly each day. For the calibration series, the stock solution was diluted in the mobile phase to form metformin concentrations of 0.1–20 μg/mL. The area under the curve (AUC) was set according to the concentration.

For extracting metformin from the medium samples, 250 μL from each sample were transferred to a 1.5 mL tube. In addition to that, an untreated medium sample was mixed with 10 μL of a 1 mg/mL metformin solution as a control. Subsequently, the protein contained in the cell culture medium was precipitated by adding 250 μL of a 1 mM silver nitrate solution (Merck KGaA). The samples were mixed for 5 min using an IKA Vibrax VXR basic shaker (IKA-Werke GmbH & Co. KG, Staufen, Germany) and centrifuged for 5 min at 4°C and 20,000 xg. The supernatant of 300 μL was transferred to the 1.1 mL HPLC tubes (Wicom Germany GmbH, Heppenheim, Germany) to be measured.

For extracting metformin from the cell pellets, samples were transferred to 1.5 mL tubes. As a control, an untreated cell pellet, that was incubated over the same amount of time and under the same conditions, was mixed with 10 μL of the 1 mg/mL metformin stock solution, the treated samples were mixed with 10 μL distilled water and then briefly vortexed. Afterwards, 300 μL of the mobile phase were added to the respective samples and the samples were shaken at 2500/min for 5 min. To disrupt the cells, the samples were placed in the ultrasonic bath Emmi -20HC (Emag Technologies Inc., Ann Arbor, MI, USA) for 15 min. Additionally, the samples were again vortexed and then centrifuged for 5 min at 4°C and 20,000 xg. After centrifugation, the supernatant of 300 μL was transferred to a 1.1 mL HPLC tube (Wicom Germany GmbH).

### Mitochondrial ROS production

Mitochondrial ROS production was determined by staining mitochondrial-derived ROS using 4 μM MitoSOX (Invitrogen, Thermofisher Scientific Inc., Waltham, MA, USA). Cells were seeded in 96 well-plates (TPP, Faust Lab Science GmbH) with a cell density of 7,500 cells/well. The cells were exposed to 1 mM metformin for 48 or 96 h. After fixation with 4% paraformaldehyde, the wells were washed with Hank’s Balanced Salt Solution (HBSS) containing calcium and magnesium. After staining in accordance with the manufacturer`s instructions for 15 min, cell nuclei were labeled with DAPI 1/1000 (Merck KGaA) for 5 min. The fluorescence intensity was measured using a Synergy 2 plate reader (BioTek Instruments GmbH). Total cell fluorescence was analyzed and normalized to cell counts.

### Caspase-3/7 activity in Adcarc1258, TCC1506, and K9UtMyo1

To determine the activity of caspase-3 and -7 the cells were seeded in 96 well-plates (TPP, Faust Lab Science GmbH) with a cell density of 7,500 cells/well. After an adherence period of 12 h the cells were incubated with 1 mM metformin for 48 or 96 h. The caspase-3 and -7 activity was measured after exposure to the Caspase-Glo® 3/7 Assay (Caspase-Glo® 3/7 Assay, Promega) in accordance with the manufacturer`s instructions for cells cultured in a 96 well-plate after 2 h. The luminescence was measured with a luminescence plate reader (BioTek Instruments GmbH) and normalized to the untreated controls [54].

### Analysis of apoptosis in Adcarc1258, TCC1506, and fibroblasts

For this purpose, the cells were incubated in triplicate in six well plates with 10⁵ cells per well over 12 h. Afterwards, the cells were treated with 1 mM metformin for 48 or 96 h. After exposure, the cells were detached with TrypLE (Gibco™, Thermo Fisher Scientific GmbH) and centrifuged together with the supernatant containing the necrotic and non-adherent cells at 160 x-g for 10 min. The cell pellet was resuspended in 500 μL binding buffer and passed through a 70 μm filter (pluriSelect Life Science UG, Leipzig, Germany). Apoptotic and necrotic cells were stained with 2.5 μL Annexin (Annexin V-FITC Detection kit Plus PromoCell). Apoptotic fractions of cells were quantified using the MACSQuant®Analyzer 10 (Miltenyl Biotec B.V. & Co. KG, Bergisch Gladbach, Germany) counting 10,000 events per sample. Data analysis was performed with FlowJo 10.3 software (FlowJo, LLC, Ashland, OR, USA). To exclude cellular debris and to set the gates, a population of apoptotic cells was generated using a 10 min stay in a 45°C water bath, and compared with untreated cells.

### Statistics

Statistical analysis of data was performed with Microsoft® Excel 2013 and SAS Enterprise Guide 7.1 (SAS Institute Inc., Cary, NC, USA). The data were expressed as mean ± standard deviation (SD). The variation analysis for determining the significant differences between single treatment, combination treatment-, and untreated cells was performed with Dunnett’s test for multiple comparisons. Additionally, Student`s t-tests were performed for comparison between 48 and 96 h, and between Adcarc1258 and TCC1506. Significance levels were set at 95% (* = p<0.05), 99% (** = p<0.01), and 99.9% (*** = p<0.001), respectively. If not described otherwise, all experiments were performed three times independently.

## Results

### Metformin alone had a higher impact on neoplastic cell division *in vitro* than DCA

**Fig 1:**
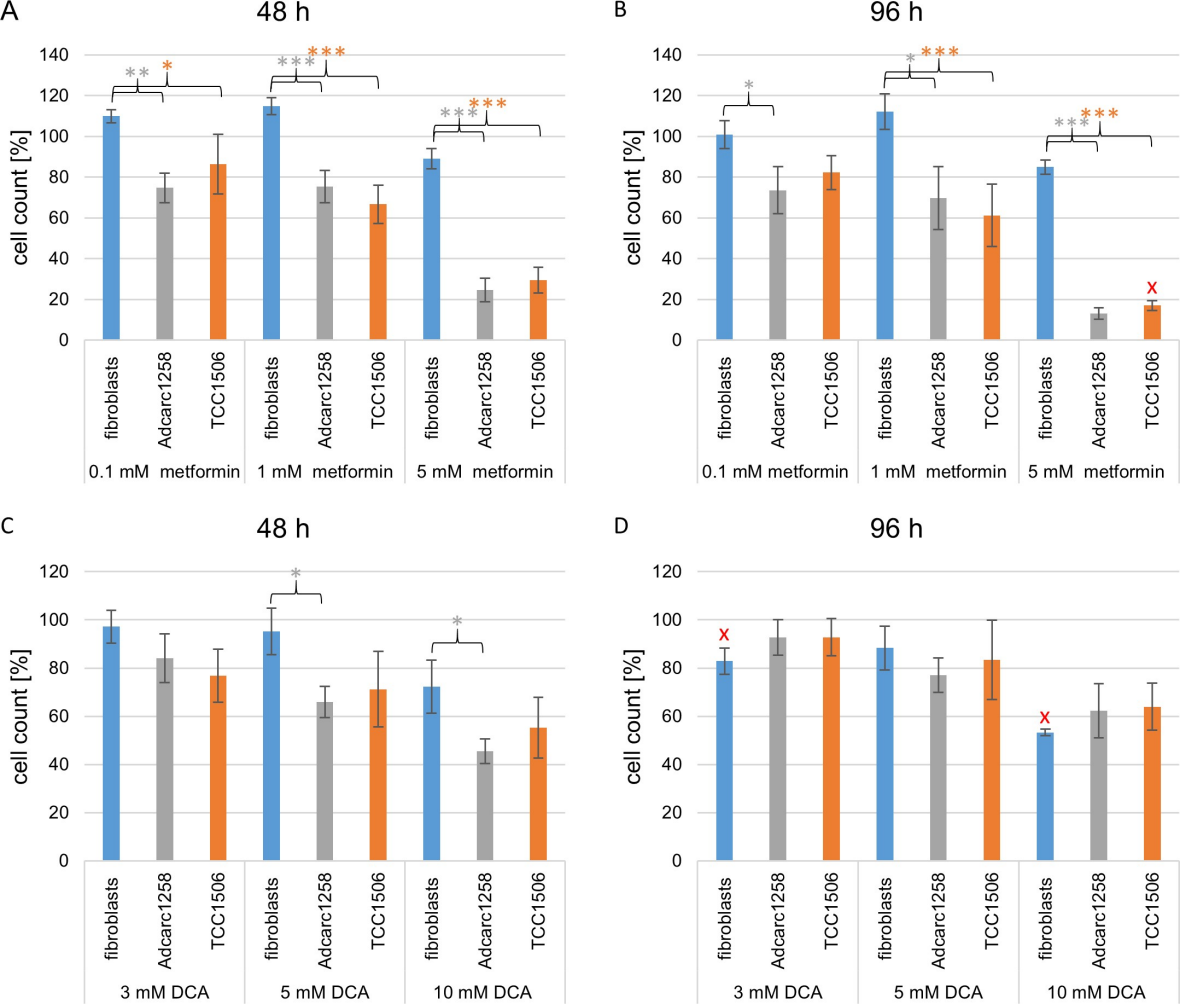
Cell count of fibroblasts, Adcarc1258 and TCC1506 after single metformin application (A and B) and single DCA application (C and D) over 48 h (A and C) and 96 h (B and D). An untreated control of the respective cell line served as a 100% reference. Means and standard deviation are displayed, n = 3. Gray asterisks: significance difference between fibroblasts and Adcarc1258. Orange asterisks: significant difference between fibroblasts and TCC1506. Red x: significant difference to the respective value at 48 h. significance is set at p* <0.05, p**<0.01 and p***<0.001, respectively.

Metformin as a single treatment (1 mM, and 5 mM) reduced the cell counts significantly (p<0.05) of the tumor cell lines Adcarc1258 and TCC1506 at 48 h and 96 h without affecting fibroblasts (Fig 1A and 1B). In contrast, the impact of DCA on cell division was lower compared to metformin, but similar between immortalized neoplastic cells and fibroblasts (Fig 1C and 1D). We observed an initial reduction in the amount of Adcarc1258 cells compared to fibroblasts 48 h after treatment with DCA (5 and 10 mM). However, this significant reduction was no longer evident after 96 h treatment. Moreover, the negative effect of DCA on the fibroblasts amount was dose and time-dependent. Thus, a longer exposure (96 h) to 3 and 10 mM DCA significantly reduced the amount of fibroblasts compared to the effect of the same doses after 48 h exposure (Fig 1D).

### DCA and metformin combined reduced cell proliferation of immortalized neoplastic cells

**Fig 2:**
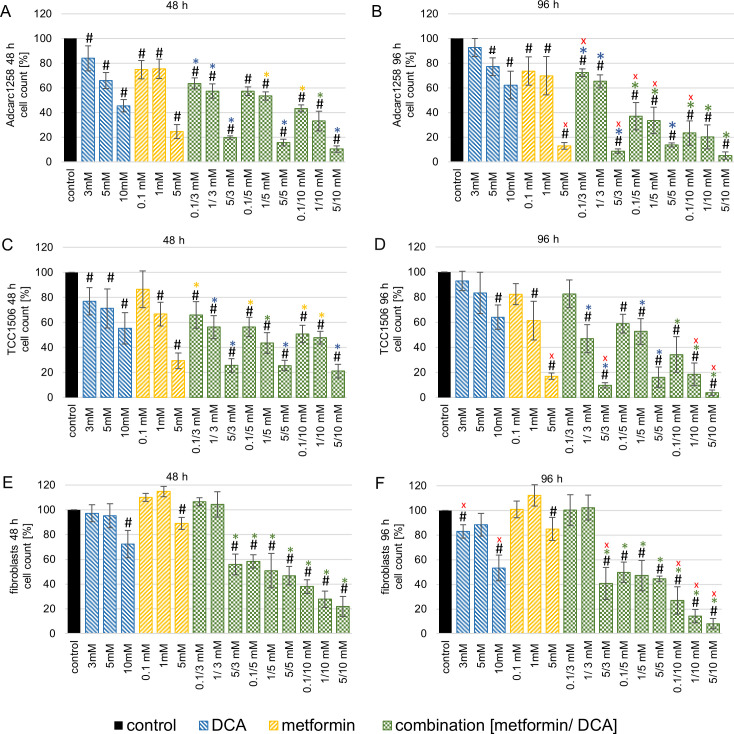
Cell count of Adcarc1258 (A and B), TCC1506 (C and D and, fibroblasts (E and F) after exposure to DCA, metformin or the combination (metformin + DCA) over 48 h (A, C, and E) and 96 h (B, D, and F). All values are expressed as a percentage of the untreated control cells of the respective cell line. Means and standard deviations are displayed, n = 3. Blue asterisks: significant difference between combination and respective single DCA application. Yellow asterisks: significant difference between combination and respective single metformin application. Green asterisks: significant difference between combination and the respective single application of DCA and metformin. Red x: significant difference referred to the respective value after 48 h exposure. Black number signs: significant difference in comparison to the control. Significance is set at p <0.05.

The combined treatment with DCA and metformin induced a time and dose-dependent reduction in the cell counts of tumor cell lines and fibroblasts (Fig 2). Moreover, the prostate adenocarcinoma cell line (Adcarc1258) was especially sensitive to the combined treatment. After 96 h, most combinations including 5 mM or 10 mM DCA reduced the cell count of Adcarc1258 compared to single treatment with the corresponding doses of DCA and metformin (Fig 2B). Furthermore, 0.1–5 mM metformin single application and 5–10 mM DCA single application decreased the cell count significantly compared to the untreated control of Adcarc1258. The combined application with 3 mM DCA reduced the cell count significantly (p<0.05) compared to the single 3 mM DCA application. However, the cell count was not decreased compared to the respective single metformin administration for 48 h and 96 h (Fig 2A and 2B). The combined application of 1 mM metformin and 5 mM DCA for 48 h reduced the cell counts significantly (p<0.05) compared to the single 1 mM metformin application, but not compared to the single 5 mM DCA application (Fig 2A). After 96 h of exposure, the cell number decreased significantly (p<0.05) with the combination of 1 mM metformin and 5 mM DCA compared to the two respective single applications.

In TCC1506 (Fig 2C and 2D), cell counts decreased compared with both single applications of DCA and metformin only in a few combinations with high dose DCA. However, single application of 1–5 mM metformin and 10 mM DCA reduced the cell count of TCC1506 compared to the untreated control of TCC1506 after 96 h (Fig 2D). After 48 h, the cell number decreased significantly (p<0.05) with the combination of 0.1 mM metformin and 3 mM DCA compared to the control and 0.1 mM metformin single application, but not compared to the 3 mM DCA application (Fig 2C). After 96 h, this combination no longer had any effect on TCC1506 (Fig 2D). The cell number decreased significantly in the combinations with 3 mM DCA and 1 mM or 5 mM metformin for 48 h and 96 h of exposure. The combination of 3 mM DCA and 5 mM metformin reduced the cell number significantly (p<0.05) at 96 h compared to the effect of the same combination at 48 h.

In the fibroblasts (Fig 2), the cell counts decreased compared to DCA and metformin single application for most of the DCA and metformin combinations. All combinations except 0.1–1 mM metformin with 3 mM DCA concentration affected the fibroblast cell counts at both time points. In addition, the single application of metformin 0.1–1 mM had no effect on the fibroblast cell count. In contrast, single application of 3 mM DCA for 96 h significantly reduced the fibroblast cell number compared to the untreated control and 48 h exposure.

### Evaluation of DCA and metformin synergistic effects on metabolic activity

**Fig 3:**
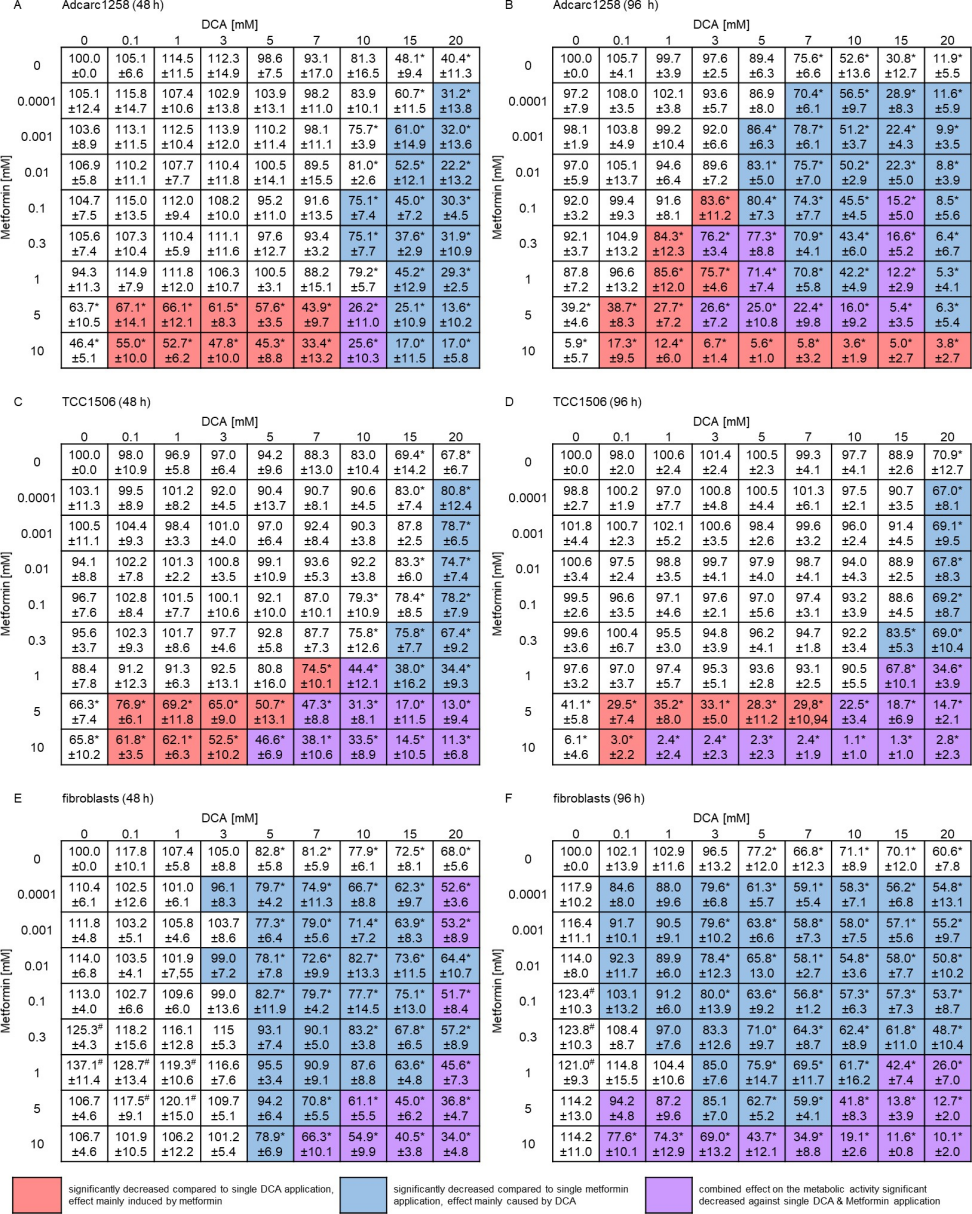
Influence of DCA, metformin or the combination of both agents on the metabolic activity of Adcarc1258 (A and B), TCC1506 (C and D), and fibroblasts (E and F) over 48 h (A, C, and E) and 96 h (B, D, and F). Effect on the metabolic activity of various concentrations of DCA (first row from above), and metformin (first left-hand column) in single application, and both agents combined (all columns and rows dose intersections). All values are expressed as percentage of the metabolic activity of untreated control cells (top left cell). Pink cells: significant difference between the respective DCA single application and the combined treatment (DCA + metformin). Blue cells: significant difference between the respective metformin single application and the combined treatment (DCA + metformin). Purple cells: significant difference between both respective single agent applications and the combined treatment (DCA+ metformin). Asterisk: significantly decreased metabolic activity compared to the untreated control and the treated samples. Number signs: significantly increased metabolic activity compared to the untreated control and the treated samples. Data are displayed as mean and standard deviation. Significance is set at p <0.05.

Tumor cells showed a significantly reduced metabolic activity after 48 h of exposure to 5 mM and 10 mM metformin alone or in combination with DCA. Metformin alone had no reducing effect on the metabolic activity of the fibroblasts (Fig 3). On the other hand, DCA alone or combinations with metformin including DCA doses higher than 5 mM (after 48 h) or higher than 0.1 mM (after 96 h) significantly reduced (p<0.05) the metabolic activity of the fibroblasts (Fig 3). Moreover, the tumor cell line Adcarc1258 showed a dose-dependant, and exposure time-dependent inhibition of the metabolic activity after 96 h treatment with combinations, even at lower doses (0.1 mM) of metformin. However, an exposure time-dependent reduction in metabolic activity was not detectable for the tumor cell line TCC1506 when comparing single DCA exposure after 48 h and 96 h. According to Bliss analysis (supplementary material), positive Bliss values of the two substances on the metabolic activity of Adcarc1258 resulted for the majority of the dose combinations. In the cell line TCC1506, positive Bliss values mainly occurred at the higher dose combinations with 10 mM DCA after 48 h. For the longer exposure time of 96 h, positive Bliss values were also present for TCC1506 in most dose combinations. In the case of fibroblasts, almost exclusively positive Bliss values in the Bliss analysis resulted after 48 and 96 h.

**Fig 4:**
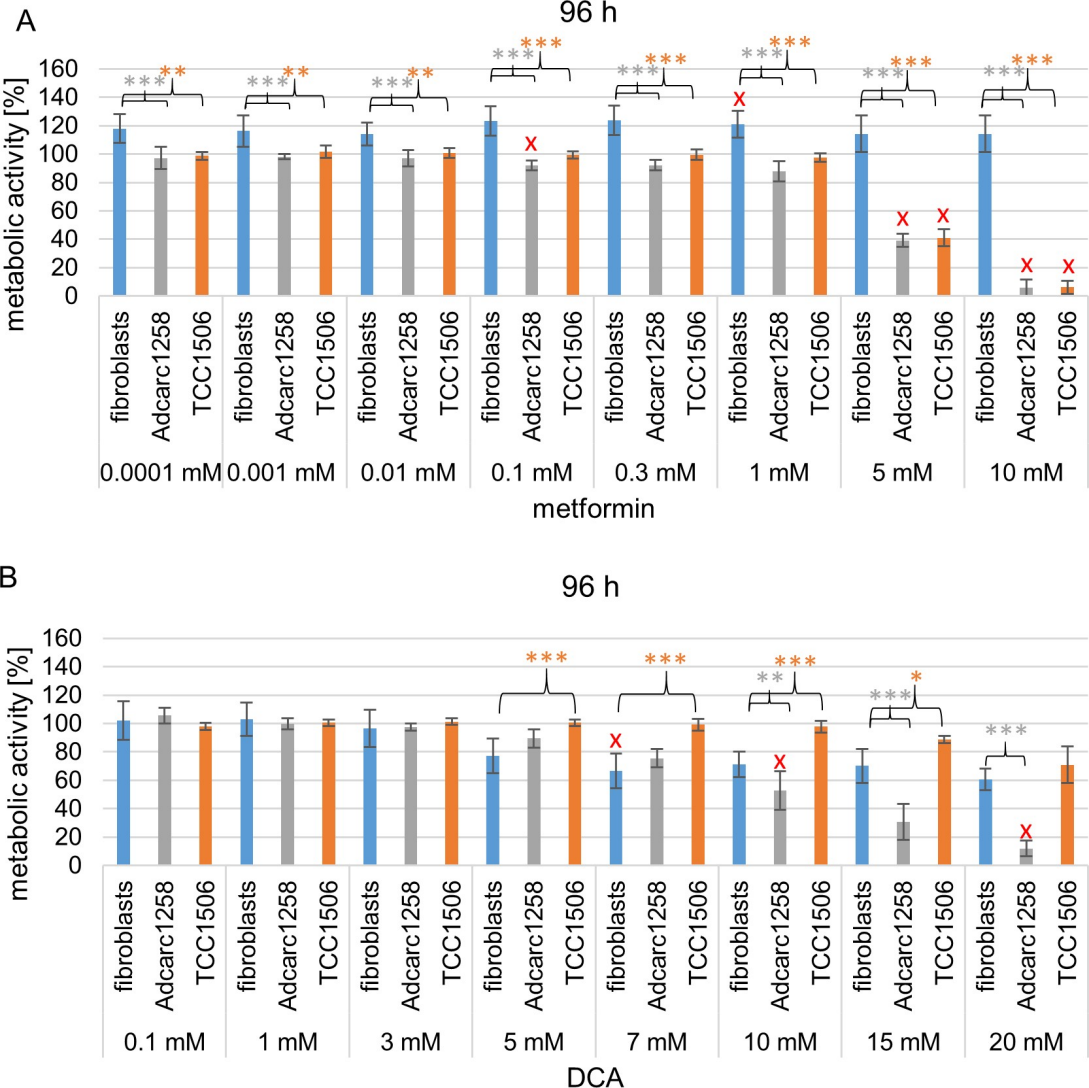
Metabolic activity of fibroblasts, Adcarc1258, and TCC1506 after exposure to increasing metformin (A) or DCA (B) concentrations over 96 h. An untreated control of the respective cell line served as a 100% reference. Means and standard deviation are displayed, n = 6. Gray asterisks: significance difference between fibroblasts and Adcarc1258. Orange asterisks: significant difference between fibroblasts and TCC1506. Red x: significant difference to the respective 48 h value. Significance is set at p* <0.05, p**<0.01 and p***<0.001, respectively.

Furthermore, after comparing the metabolic activity of tumor cells and fibroblasts exposed to metformin or DCA as single agents at 48 h and 96 h (Fig 4), no difference on the metabolic activity of the cells was observed for most DCA doses at 48 h. On the other hand, an exposure time of 96 h to 10–20 mM DCA significantly reduced the metabolic activity of Adcarc1258 compared to fibroblasts. The metabolic activity of TCC1506 compared to that of the fibroblasts was increased at 5, 7, 10, and -15 mM DCA, respectively. After 48 h exposure to metformin (doses up to 0.3 mM), a reduction in the metabolic activity of the tumor cells compared to the metabolic activity of the fibroblasts was observed (Fig 4). After 96 h exposure, all metformin doses employed significantly reduced the metabolic activity of tumor cells compared to fibroblasts. For both substances, an enhanced inhibition of metabolic activity for tumor cells could be detected for the longer exposure interval of 96 h compared to 48 h. Furthermore, metformin increased the metabolic activity of fibroblasts after 96 h exposure period compared to 48 h exposure period.

### Metformin affected mitochondrial ROS production

**Fig 5:**
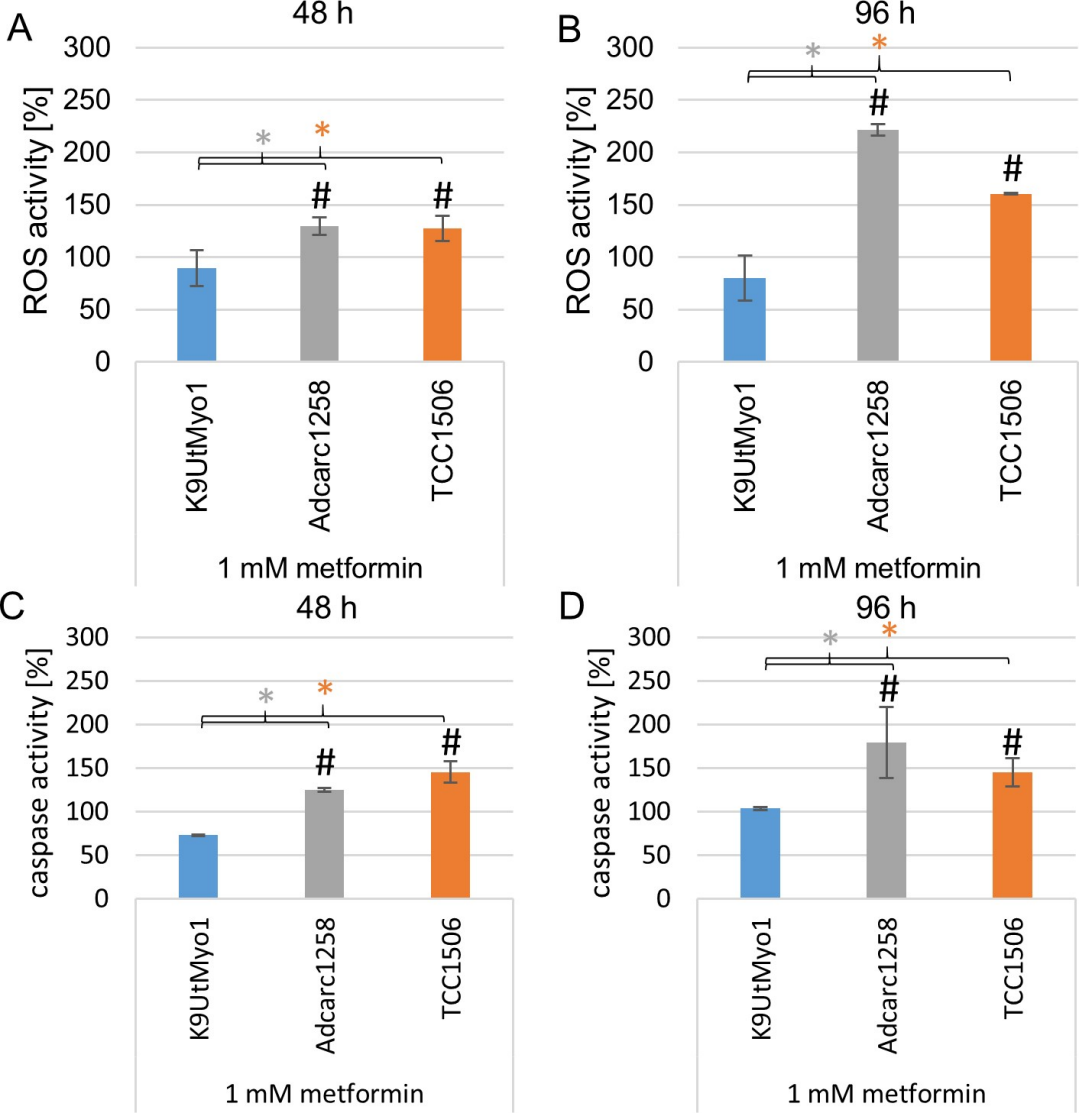
Mitochondrial ROS production (A and B) and caspase-3/7 activity (C and D) of K9UtMyo1, Adcarc1258, and TCC1506 after single 1 mM metformin application over 48 h (A, C) and 96 h (B, D). An untreated control of the respective cell line served as a 100% reference. Means and standard deviation are displayed, n = 3. Gray asterisk: significant difference between K9UtMyo1 and Adcarc1258. Orange asterisk: significant difference between K9UtMyo1 and TCC1506. Black number signs: significant difference in comparison to the control. Significance is set at p <0.05.

The mitochondrial-derived ROS production increased significantly after exposure to 1 mM metformin in both investigated cancer cell lines over 48 and 96 h compared to an untreated control and K9UtMyo1 (Fig 5A and 5B).

### Metformin-induced increased caspase-3/7 activity and apoptosis in canine lower urinary tract tumor cell lines

**Fig 6:**
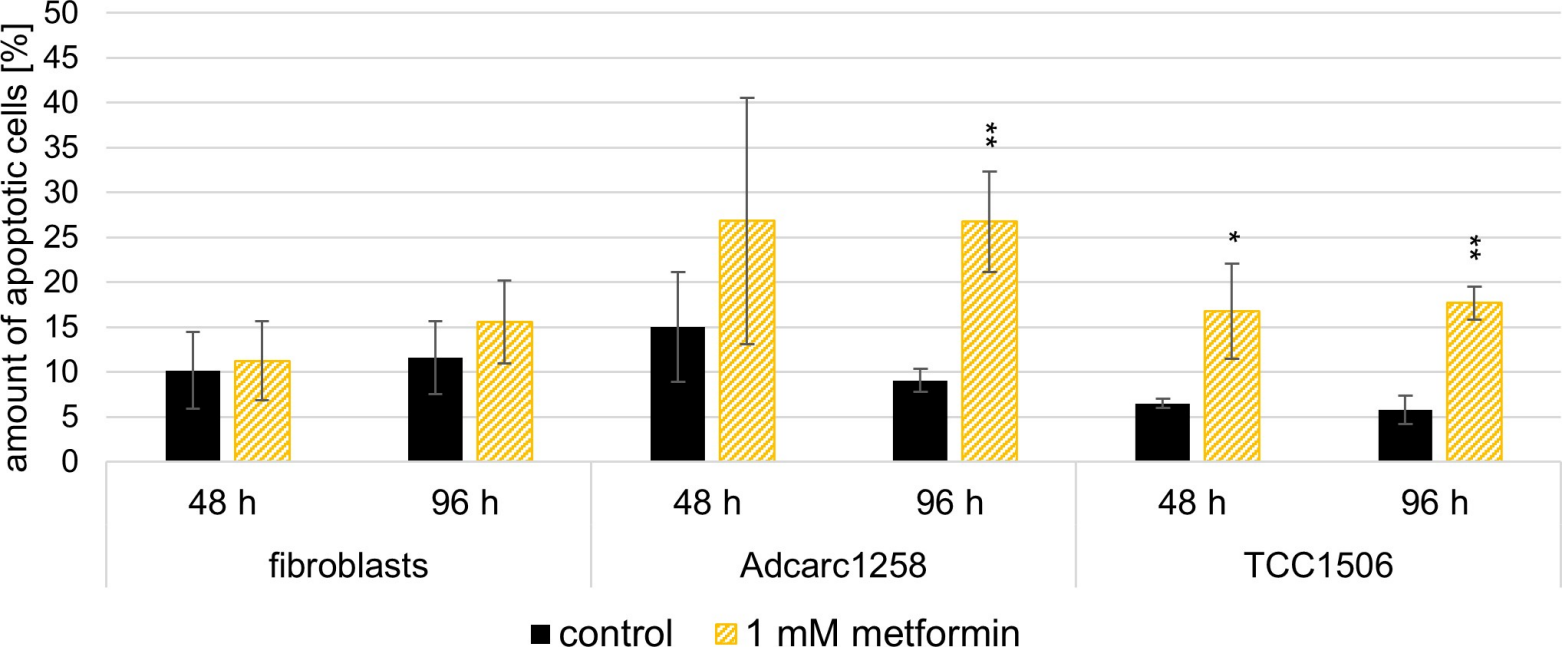
Apoptosis induction in fibroblasts, Adcarc1258, and TCC1506 after exposure to 1 mM metformin compared to an untreated control of the respective cell line over 48 and 96 h. Means and standard deviation are displayed, n = 3. Black asterisks: significant difference between the respective untreated control and treatment with 1 mM metformin. Significance is set at p* <0.05, p**<0.01 and p***<0.001, respectively.

The exposure to 1 mM metformin over 48 h and 96 h increased the caspase-3/7 activity of Adcarc1258 and TCC1506 compared to control and as well as K9UtMyo1 (Fig 5C and 5D). After 48 h exposure to 1 mM metformin, the TCC1506 cells showed an increased amount of apoptotic cells (Fig 6). The proportions of apoptotic cells increased in TCC1506 and Adcarc1258 after exposure to 1 mM metformin, whereas 1 mM metformin had no effect on the apoptotic amount of fibroblasts over a 48 or 96 h exposure period (Fig 6 and S3 Fig).

### Fibroblasts and myocytes detection

**Fig 7:**
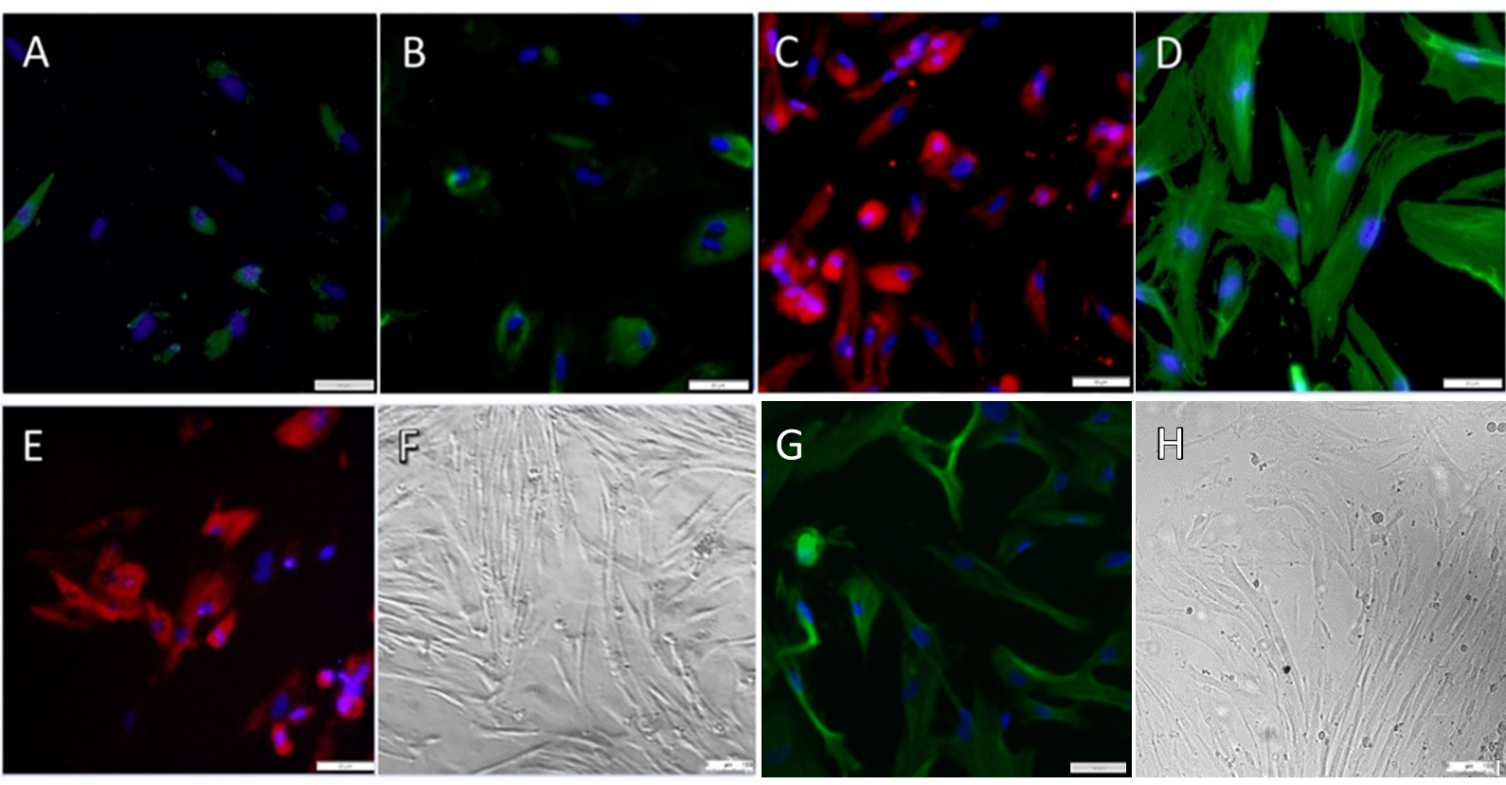
Immunofluorescence analysis of isolated fibroblasts and K9UtMyo1, (A) fibroblasts collagen I (green), (B) fibroblasts collagen III (green), (C) fibroblasts collagen VI (red), (D) fibroblasts vimetin (green), (E) fibroblasts fibronectin (red) and (G) K9UtMyo1 SMA (green) (A-E and G) counterstained cell nuclei (DAPI, blue); (F) morphology of primary cell culture of canine oral mucosa fibroblasts (H) morphology of K9UtMyo1 by light microscopy (at a magnification of 100x).

In the immunofluorescence analysis, the isolated fibroblasts were positive for collagen I, III, VI, vimentin, and fibronectin (Fig 7). Vimentin was evenly distributed in the cytoplasm of the isolated spindle-shaped fibroblasts, whereas fibronectin was localized extracellularly. Collagen I, III, and VI staining was perinuclear. The isolated K9UtMyo1 stained vimentin and smooth muscle actin (SMA) positive.

### Quantitative measurement of metformin in cell culture medium and cell pellet by HPLC

**Fig 8:**
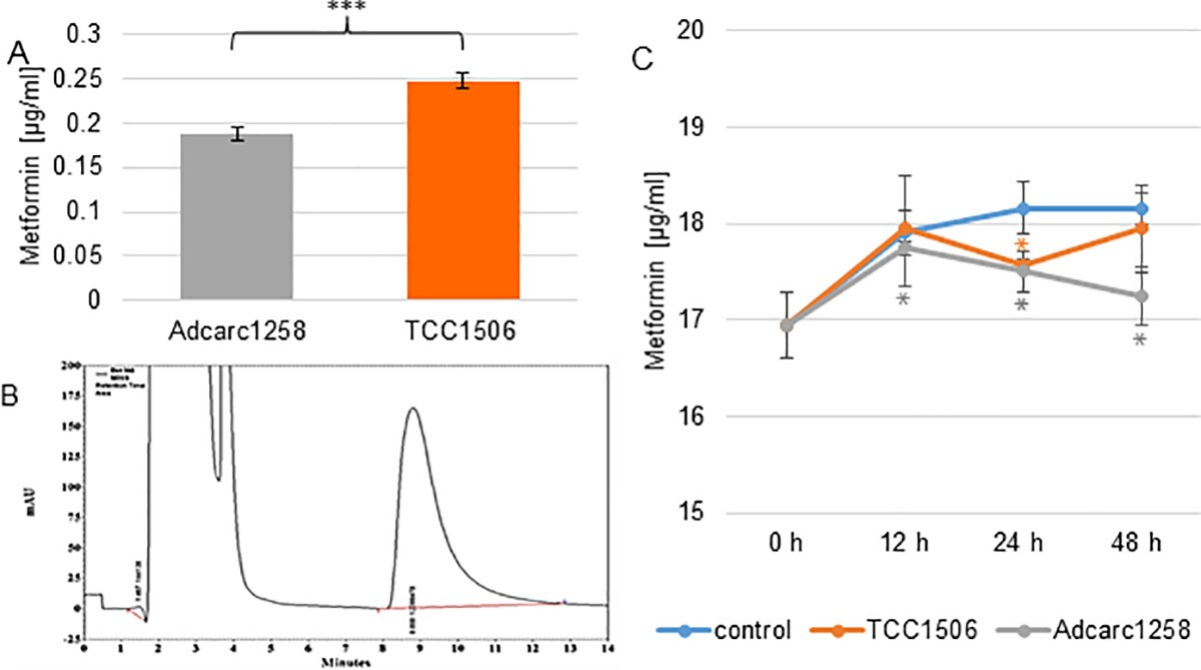
Metformin content in cell pellets of Adcarc1258 and TCC1506 after exposure to 0.1 mM metformin over 48 h (A). Typical chromatogram of metformin 10 μg/mL in cell culture media (B). Metformin content in cell culture medium supernatant of TCC1506 (orange line) and Adcarc1258 (gray line) in comparison to a control (blue line) incubated with 0.1 mM metformin parallel to the tumor cells without cells (C). Means and standard deviation are displayed, n = 3. Black asterisks: significant difference between Adcarc1258 and TCC1506 (A). Gray asterisks: significant difference between the control and Adcarc1258 (C). Orange asterisks: significant difference between control and TCC1506 (C). Significance is set at p* <0.05, p**<0.01 and p***<0.001, respectively.

Metformin concentration in cell culture medium and in the cell pellet was calculated with reference to the calibration curve using the HPLC method. The calibration curves for the mobile phase as well as for the medium were linear over the range from 1 to 200 μM, with a regression coefficient of 0.99. HPLC chromatogram of metformin was detected at a retention time of 8.8 min. The lower limit of quantification (LLOQ) for metformin in the mobile phase was 0.17 μg/mL and the lower limit of detection (LLOD) was 0.09 μg/mL. In the cell culture medium, the LLOQ was 1.4 μg/mL and the LLOD 0.7 μg/mL. After 24 h, the metformin concentration in the metformin-spiked cell culture medium incubated in the presence of either Adcarc1258 or TCC1506 was decreased compared with cell-free control. This effect was further observed after 48 h in Adcarc1258, while the metformin concentrations in the medium incubated with or without TCC1506 cells did not differ after 48 h (Fig 8C). The metformin content measured after 12 h and 24 h in the cell pellet of the TCC1506 was just above the LLOQ of the mobile phase and increased after 48 h exposure time. Adcarc1258 only achieved metformin contents in the cell pellet in the area of the LLOQ of the mobile phase after 48 h exposure time.

## Discussion

*In vitro* studies showed that canine prostate adenocarcinomas and transitional cell carcinomas responded to DCA treatment with decreased proliferation rates, increased pyruvate oxidation, and mitochondrial activity [25]. To reduce the required DCA dose and associated side effects and to avoid resistance development, the combination therapy with a synergistic substance is a promising approach in cancer therapy [55]. In vitro studies demonstrated a synergistic effect of DCA and metformin in human breast cancer cells [13], human ovarian cancer cells [14], Lewis lung carcinoma [56], and murine glioblastoma cells [15]. Both substances are easy to synthesize and metformin is already approved as therapeutic for human medicine [56, 57], while DCA has been used in humans for the treating lactic acidosis [24, 58, 59]. Therefore, the combination of DCA and metformin is less critical regarding the side effect potential than conventional cytostatics [14, 18, 56]. The present study investigates the effect of metformin, DCA, and the combination of both substances on canine carcinoma cells in comparison to isolated canine fibroblasts and K9UtMyo1.

Although the tumormicroenvironment (TME) and the cancer-associated fibroblasts (CAFs) should also be considered [60, 61], the isolated fibroblasts are normal fibroblasts. The fibroblasts and K9UtMyo1 are supposed to represent the healthy tissue cells and had no direct or indirect tumor cell contact. In contrast, CAFs are altered by complex interactions with tumor tissue and are clearly different from normal fibroblasts [62, 63]. As a healthy tissue cell model, the fibroblasts and K9UtMyo1 should help to distinguish an effect on tumor cell metabolism from an effect on healthy cells [64, 65]. Furthermore, fibroblasts and K9UtMyo1 were studied in comparison to the two tumor cell lines to minimize the individual and cell type- specific heterogeneity described for metformin [66]. In general, the goal of this combination therapy should be a specific inhibition of tumor cell metabolism. Thus, it should be associated with fewer side effects on healthy tissue cells than, for example, cytostatics [64]. The results show an inhibition of metabolic activity and cell number as well as an increase in the apoptotic cell fraction of tumor cell lines studied after metformin exposure. On the one hand, metformin induces cell cycle arrest in human prostate cancer cells through a decrease in Cyclin D1 expression and an upregulation of *REDD1* [34, 67, 68]. On the other hand, metformin increases apoptosis induction in tumor cells [33, 69] or a combination of apoptotic and antiproliferative effects reduces the tumor cell number [70]. However, increased apoptosis is not sufficient to explain the cell count reduction in the tumor cells on its own. Therefore, a combination of apoptosis induction and inhibition of cell proliferation is plausible. In contrast to tumor cell lines, metformin exposure in isolated fibroblasts confirmed by immunocytochemistry showed an increasing effect on metabolic activity. An increased metabolic activity of fibroblasts after metformin exposure was reported by Qin et al. [71]. In accordance with these findings, targeting mitochondrial respiration by overexpressing mitochondrial uncoupling proteins leads to a reduced cancer cell viability, but increased high-energy production in fibroblasts [72, 73]. Furthermore, metformin exposure had little effect on the number and no effect on apoptosis induction of fibroblasts. Additionally, metformin exposure had no effect on the ROS production and caspase-3/7 activity of K9UtMyo1. Metformin shows comparable antiproliferative and apoptosis-inducing effects in human oral squamous cell carcinoma [70], and no cytotoxicity on human fibroblasts [74]. Moreover, metformin increases ROS production in human pancreatic cancer cells but not in human dermal fibroblast [75]. The differential response towards metformin exposure could be explained by the selective activation of the AMPK/FOXO3a/MnSOD pathway in healthy cells but not in cancer cells [75]. Oxidative stress activates FOXO3a protein, which in turn activates ROS removal enzymes as a catalase and MnSOD [75–78]. The selective activation of the AMPK/FOXO3a/MnSOD pathway could be due to downregulation or inactivity of FOXO3a protein in different cancer cells [79–81]. On comparing the metabolic activity and the cell count, it is noticeable that the cell count already decreases after exposure to lower concentrations of the substances, whereas the metabolic activity only decreases after exposure to higher concentrations of the two substances in both tumor cell lines and fibroblasts. This difference between cell count and metabolic activity results could be explained by the enhancing effect of metformin on the metabolic activity of the remaining cells due to drug-induced changes in the mitochondrial activity [82]. For example, metformin inhibits complex-1 of the electron transport chain in the mitochondria membrane, which, in turn, might secondarily activate AMPK (AMP-activated protein kinase). AMPK is able to change the cell metabolism from anabolic to catabolic, and decreases the cellular energy status of the cancer cells [68].

To be able to draw conclusions from the *in vitro* results concerning a possible *in vivo* applicability, it is important to consider which is the maximum tolerated drug dose in the target tissue of the organism without adverse severe side effects. In a pre-prostatectomy study of human prostate cancer patients, the serum and prostate tissue concentration of metformin was determined. After 4–12 weeks of daily administration, serum concentrations increased from undetectable to 3.6 μg/mL, and prostate tissue concentrations of 0.88 to 51.2 μg/g were measured [83]. The observed pharmacokinetics of metformin in dogs seemed to be comparable to the pharmacokinetics reported for cats and humans [84]. However, the available data do not allow a maximum tolerated dose to be determined. Therefore, further studies on the tolerability of metformin in dogs are needed. Human prostate tissue concentrations can be used as an orientation to interpret the *in vitro* results. To qualify this assumption, this study investigated the kinetics of metformin in the specified cell culture medium and the amount of metformin detectable in tumor cells, and possible accumulation under cell culture conditions. The results of the quantitative measurement of metformin in cell culture medium and tumor cell pellets by HPLC may indicate a metabolism of metformin by Adcarc1258. However, which kind of metformin metabolites occurs remains unclear and requires further research. Taking these results into consideration, the dose range of metformin used in the *in vitro* experiments here covers the clinically relevant range. Another aspect concerns the inefficient vascularization of the tumor microenvironment, which leads to an undersupply of nutrients like glucose and growth factors [85]. Low glucose levels increase the cytotoxicity of metformin on cancer cells [86]. Consequently, tumor cells might be more sensitive to metformin *in vivo* than under the cell culture conditions tested in this study.

For the combination of DCA and metformin, positive Bliss values and significant differences in metabolic activity and cell count compared to both single applications were obtained in the investigated cancer cell lines, indicating a synergistic effect of the two substances on the metabolic activity and cell count. The observed synergistic effect is consistent with the results of previous combination therapy studies in different cancer cell lines [13–16]. For example, DCA cytotoxicity on murine glioblastoma cells was enhanced by metformin-induced complex-1 inhibition [15]. In addition, combining DCA with metformin increased oxidative cell damage, and consequently increased cell death of human breast cancer cells [13, 16]. Furthermore, the combination leads to the inhibition of human ovarian cancer cell growth [14]. The two substances showed a time-dependent effect when comparing the 48 h and 96 h treatment. This could be explained by the modulating effect of DCA and metformin on the energy metabolism of the cells [56, 87]. Since the microenvironment of the tumor cells changed over the longer incubation time, fewer nutrients were available for the energy production of the cells [56, 87]. For differentiated analysis, the modified checkerboard layout (see Fig 2) allowed the investigation of multiple-dose combinations. In addition to the Bliss Analysis, the significance of the combination results was calculated in comparison to the individual single application used to evaluate the biological relevance. Despite the checkerboard approach, all identified combinations with biologically relevant synergism on both tumor cell lines exceeded the maximum plasma concentration of 3 mM DCA tolerable in dogs [26]. In contrast to the study by Haugrud et. al. who were unable to demonstrate a cytotoxic effect on non-neoplastic mammary epithelial cells [13], DCA showed an inhibition of metabolic activity and cell number in the isolated fibroblasts, especially in higher dose combinations with metformin. Hence, the present data do not support a practical DCA dose reduction in the case of combination with metformin that is better tolerated by patients.

## Conclusion

The quantitative metformin measurement by HPLC, together with the results of the metabolic activity assay, cell count analysis, mitochondrial ROS production, and apoptosis assay observed in this study suggest a biologically relevant effect of metformin on canine carcinoma cells. Thus, metformin could be a suitable treatment for canine prostate adenocarcinoma and transitional cell carcinoma. However, further studies on the tolerability, mode of application, and efficacy *in vivo* are still necessary. The antiproliferative and inhibitory effects on the metabolic activity of both tumor cells and fibroblasts have been demonstrated for the combination of DCA and metformin. There is a clear antiproliferative effect of DCA on tumor cells but unfortunately, this cannot be separated from that on non-neoplastic cells. Consequently, further studies on possible combination regimes including other substances, and the evaluation of their effects on non-malignant cells are needed.

## Supporting information

S1 Table Cell line characterization. (DOCX)

S2 Table Antibodies used for fibroblast detection. (DOCX)

S1 Fig Bliss values. Bliss values of the combination of DCA and metformin calculated for the effect on the metabolic activity of Adcarc1258 (A- and B), TCC1506 (C- and D), and fibroblasts (C and- F) over 48 h (A, C, and E) and 96 h (B, D, and F). Bliss values after exposure to several doses of DCA (first top row), and metformin (first left hand column) in different combinations of DCA and metformin (all columns and rows doses intersections) Positive Bliss values are highlighted in green, Bliss values equal to zero are highlighted in yellow, negative Bliss values are highlighted in red.
(TIF)

S2 Fig Metabolic activity 48 h. Metabolic activity of fibroblasts, Adcarc1258, and TCC1506 after exposure to increasing metformin (A) or DCA (B) concentrations over 48 h. An untreated control of the respective cell line served as a 100% reference. Means and standard deviation are displayed; n = 6. Gray asterisks: significance difference between fibroblasts and Adcarc1258. Orange asterisks: significant difference between fibroblasts and TCC1506. Significance is set at p* <0.05, p**<0.01, and p***<0.001, respectively.
(TIF)

S3 Fig Representative dot plots of flow cytometry data. The three cell lines were stained with Annexin V-FITC and TO-PRO-3 iodide. Cells in area Q4 were counted as vital, cells in Q1 as late apoptotic, and in Q2 and Q3 as early apoptotic.
(TIF)

S4 Fig Representative chromatograms HPLC analysis. (TIF)

S1 File Cell count raw data. (XLSX)

S2 File Metabolic activity raw data. (XLSX)

S3 File Apoptosis raw data. (XLSX)

S4 File HPLC raw data. (XLSX)
