# Supplementary material for: Metformin and sodium dichloroacetate effects on proliferation, apoptosis, and metabolic activity tested alone and in combination in a canine prostate and a bladder cancer cell line
Source: PLoS One. 2021 Sep 27;16(9):e0257403. doi: 10.1371/journal.pone.0257403 (PMC8476037; doi:10.1371/journal.pone.0257403)
Supplement: S1 Table — (DOCX) [file pone.0257403.s001.docx]

|  | Adcarc1258 | TCC1506 |
| --- | --- | --- |
| Doubling times (DT) of cell lines in h | 21.6 | 19.9 |
| Growth behavior | Low anchoring potential, ability to form aggregates | Monolayer, cobblestone-like |
| IC50 Doxorubicin [nM]  Metabolic activity  Cell count | 352.0  85.0 | 32.0  15.5 |
| IC50 Carboplatin [µM]  Metabolic activity  Cell count | 97.7  6.6 | 39.8  9.0 |
| Significant induction of apoptosis  Doxorubicin [nM]  Carboplatin [µM] | 100  100 | 200  200 |
| Epithelial marker  Pan-CK  CK7  CK8/18  E-Cad | -  -  -  - | +  -  +  + |
| Mesenchymal marker  Vimentin  Calp | +  + | -  + |

**S1 Table. Cell line characterization**

Key aspects of the investigated cancer cell lines Adcarc1258 and TCC1506. For extensive cell line characterization see Packeiser et al (2020). Immunohistochemical staining of cell lines + = positive; - = negative.
