# Supplementary material for: Metformin and sodium dichloroacetate effects on proliferation, apoptosis, and metabolic activity tested alone and in combination in a canine prostate and a bladder cancer cell line
Source: PLoS One. 2021 Sep 27;16(9):e0257403. doi: 10.1371/journal.pone.0257403 (PMC8476037; doi:10.1371/journal.pone.0257403)
Supplement: S2 Table — (DOCX) [file pone.0257403.s002.docx]

**S2 Table. Antibodies used for fibroblast detection**

| antibody | type | clone | dilution | company |
| --- | --- | --- | --- | --- |
| fibronectin | rabbit anti-human | monoclonal  ab32419 | 1/100 | Abcam, Cambridge, UK |
| vimentin | mouse anti-human | monoclonal  ab8069 | 1/200 | Abcam, Cambridge, UK |
| collagen I | mouse anti-human | monoclonal  ab6308 | 1/100 | Abcam, Cambridge, UK |
| collagen III | mouse anti-human | monoclonal  ab6310 | 1/100 | Abcam, Cambridge, UK |
| collagen VI | rabbit anti-human | monoclonal  ab6588 | 1/100 | Abcam, Cambridge, UK |
| actin (smooth muscle) | mouse anti-human | monoclonal  M0851 | 1/100 | Agilent, Santa Clara, CA, US |
| secondary antibodies | goat anti- rabbit | polyclonal | 1/250 | Cell Signaling Technology, Leiden, Netherlands |
| secondary antibodies | goat anti-mouse | polyclonal | 1/1000 | Abcam, Cambridge, UK |
