# Supplementary figures and images for: Metformin and sodium dichloroacetate effects on proliferation, apoptosis, and metabolic activity tested alone and in combination in a canine prostate and a bladder cancer cell line
Source: PLoS One. 2021 Sep 27;16(9):e0257403. doi: 10.1371/journal.pone.0257403 (PMC8476037; doi:10.1371/journal.pone.0257403)

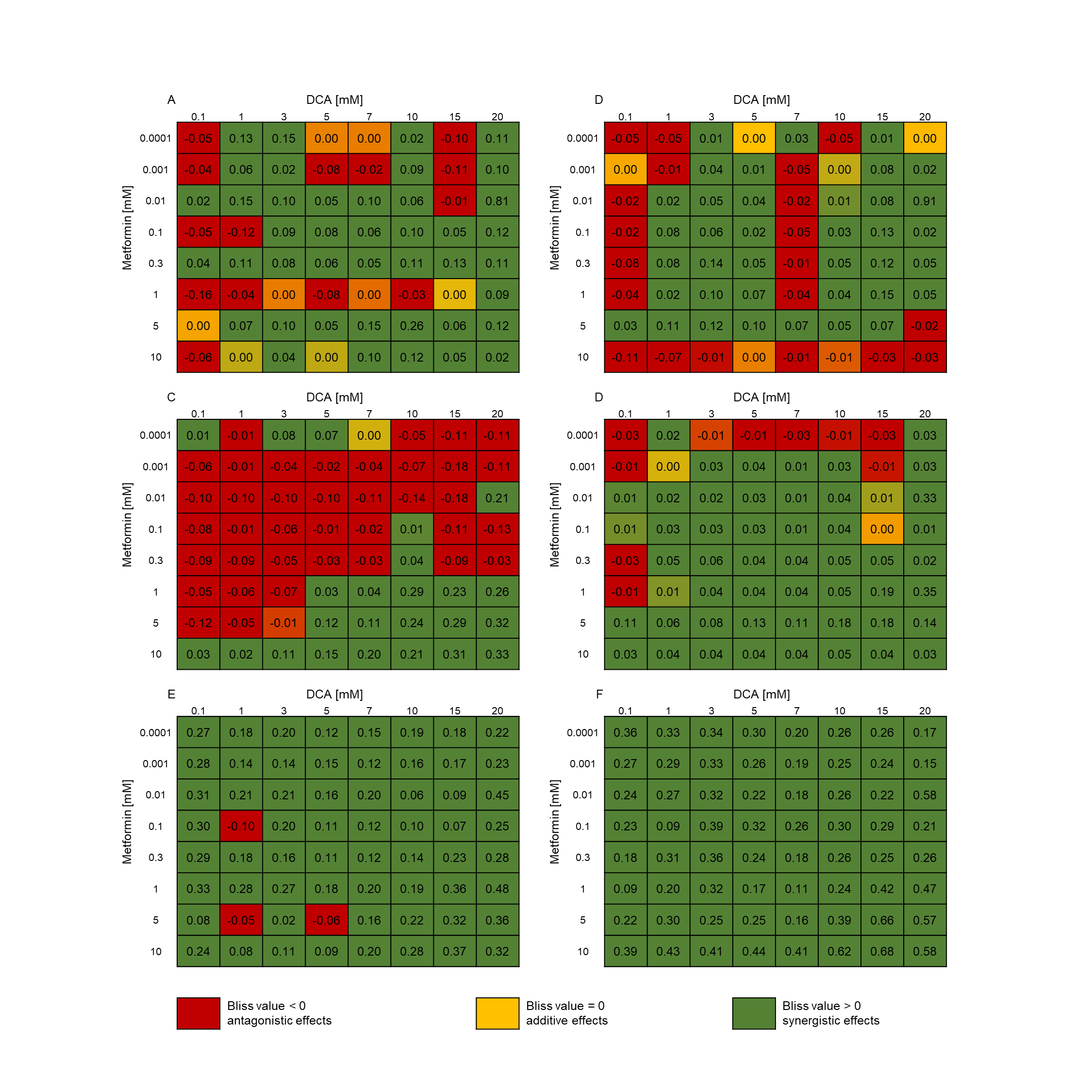

Supplement: S1 Fig — Bliss values of the combination of DCA and metformin calculated for the effect on the metabolic activity of Adcarc1258 (A- and B), TCC1506 (C- and D), and fibroblasts (C and- F) over 48 h (A, C, and E) and 96 h (B, D, and F). Bliss values after exposure to several doses of DCA (first top row), and metformin (first left hand column) in different combinations of DCA and metformin (all columns and rows doses intersections) Positive Bliss values are highlighted in green, Bliss values equal to zero are highlighted in yellow, negative Bliss values are highlighted in red. (TIF) [file pone.0257403.s003.tif]

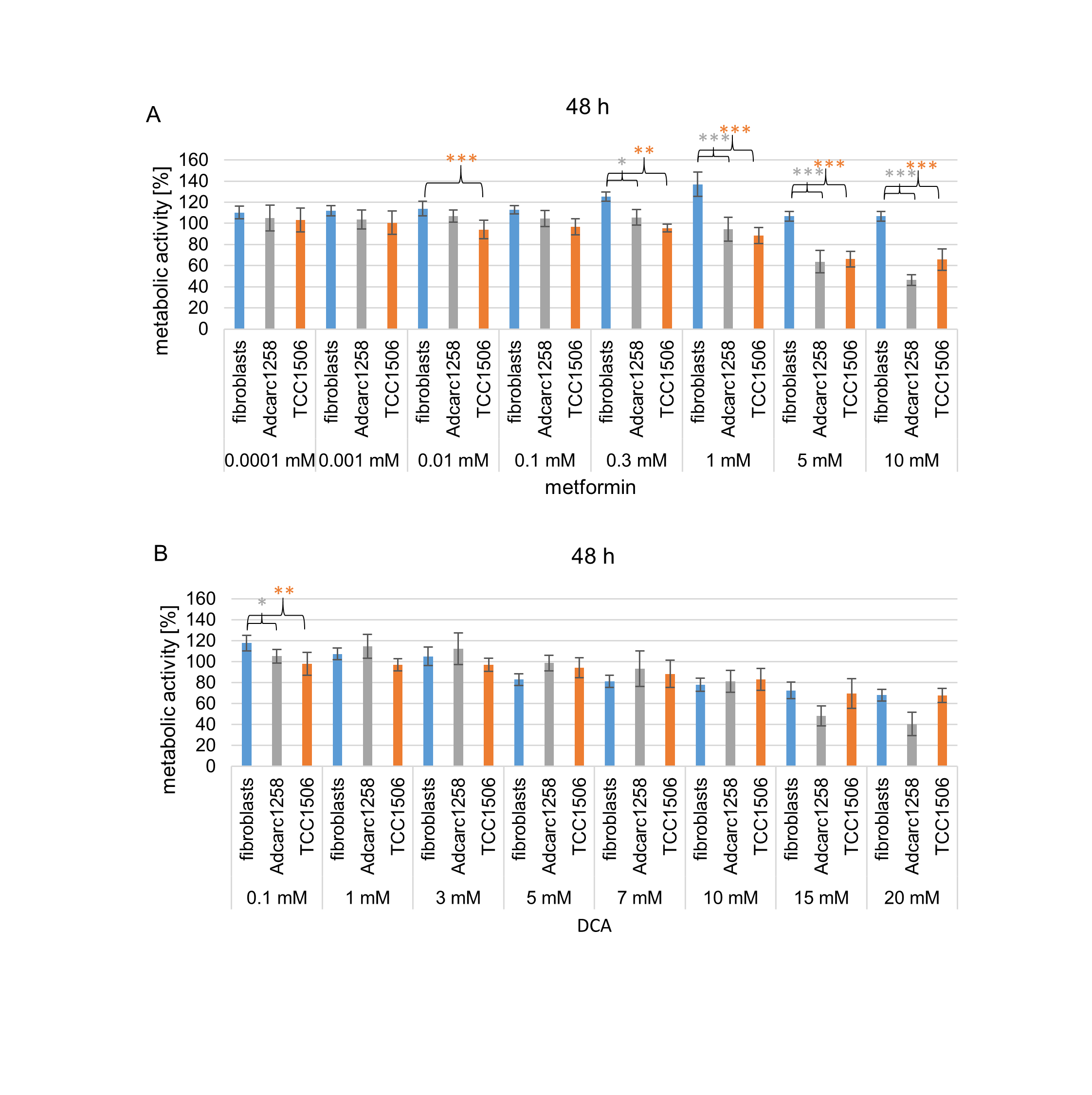

Supplement: S2 Fig — Metabolic activity of fibroblasts, Adcarc1258, and TCC1506 after exposure to increasing metformin (A) or DCA (B) concentrations over 48 h. An untreated control of the respective cell line served as a 100% reference. Means and standard deviation are displayed; n = 6. Gray asterisks: significance difference between fibroblasts and Adcarc1258. Orange asterisks: significant difference between fibroblasts and TCC1506. Significance is set at p* <0.05, p**<0.01, and p***<0.001, respectively. (TIF) [file pone.0257403.s004.tif]

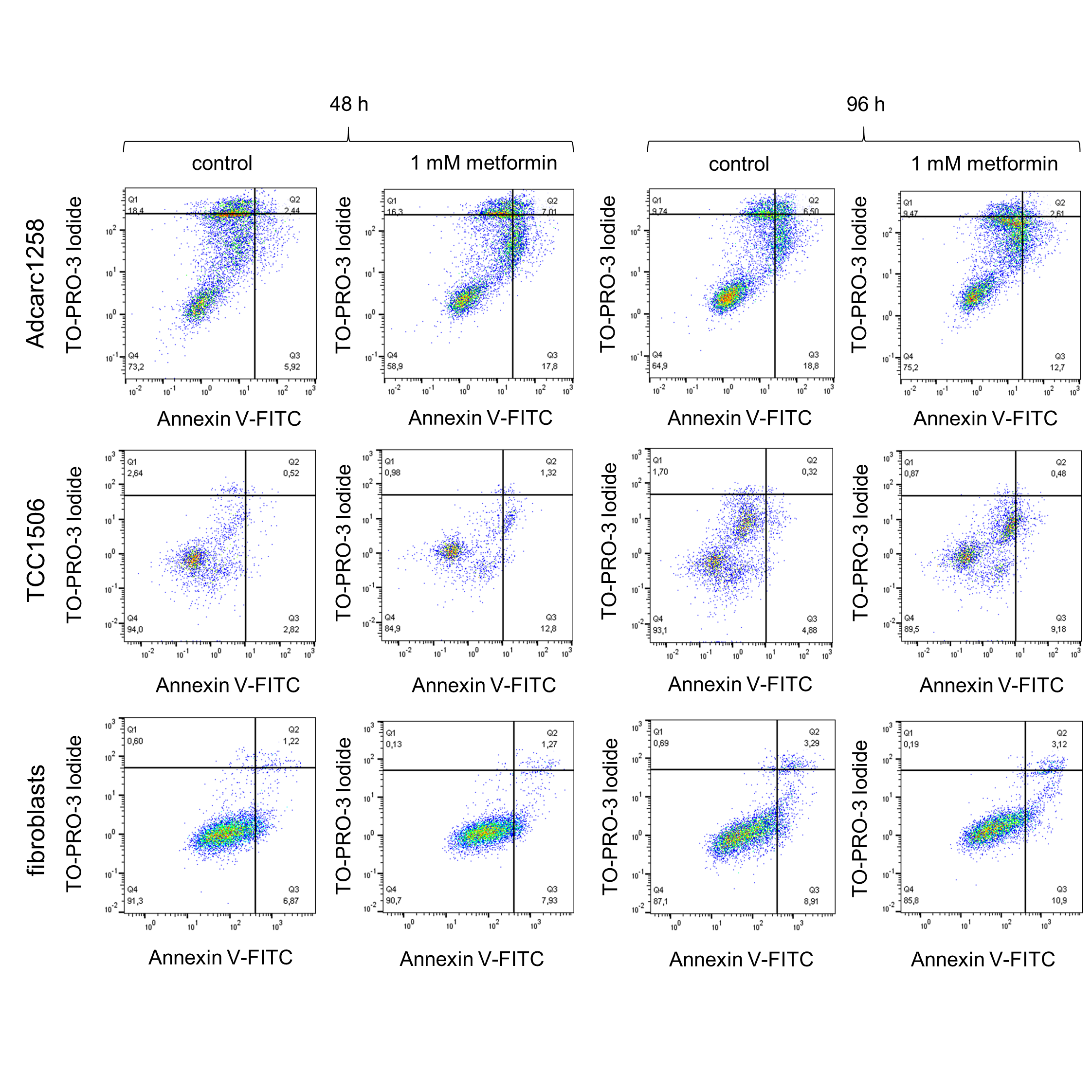

Supplement: S3 Fig — The three cell lines were stained with Annexin V-FITC and TO-PRO-3 iodide. Cells in area Q4 were counted as vital, cells in Q1 as late apoptotic, and in Q2 and Q3 as early apoptotic. (TIF) [file pone.0257403.s005.tif]

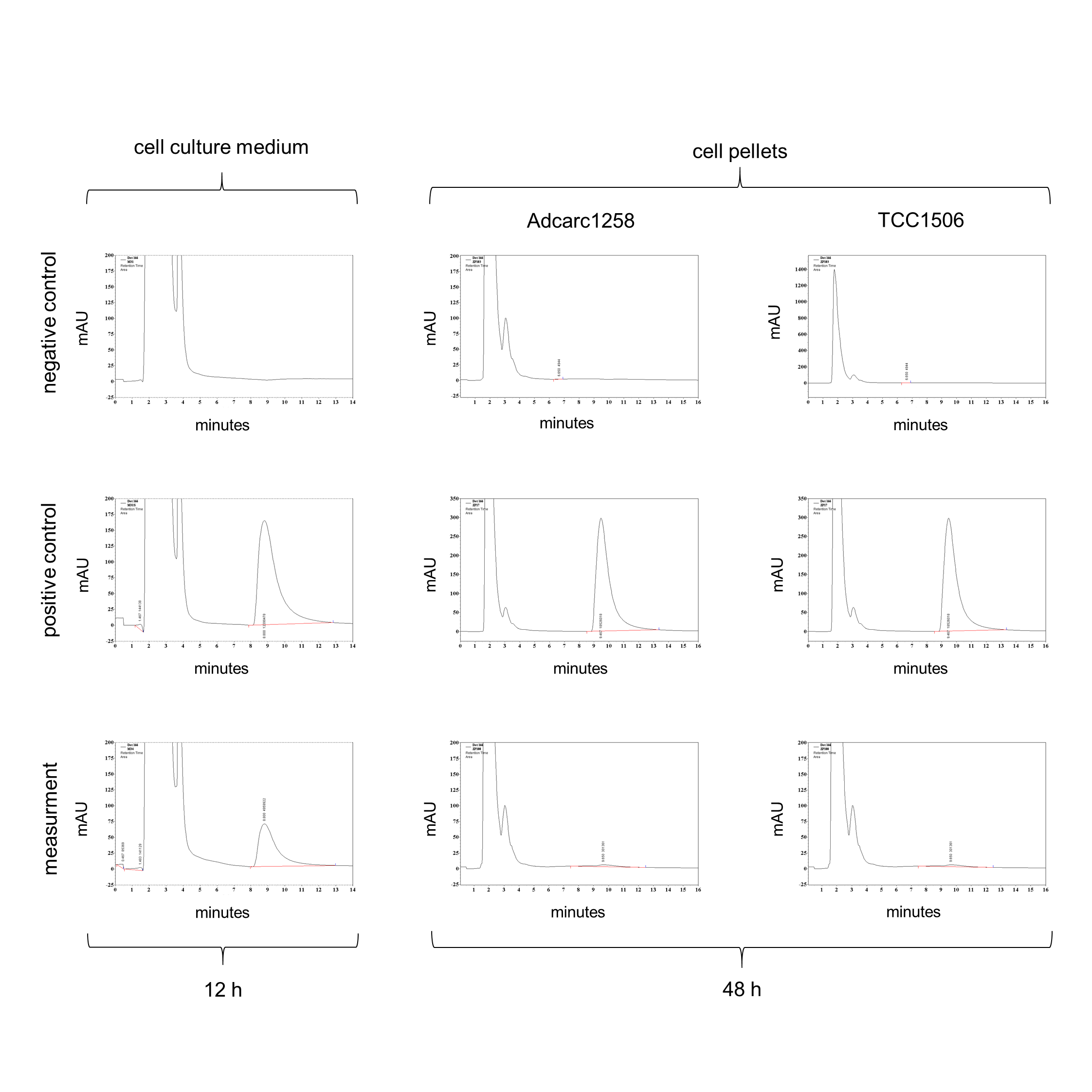

Supplement: S4 Fig — (TIF) [file pone.0257403.s006.tif]
